# Integration of single-cell and bulk transcriptomics uncovers CHST6 as a shared pathogenic driver in idiopathic pulmonary fibrosis and lung cancer

**DOI:** 10.1063/5.0289275

**Published:** 2025-12-16

**Authors:** Shengqiang Mao, Jiayi Jiang, Zhiqiang Liu, Yi Li

**Affiliations:** 1 Department of Pulmonary and Critical Care Medicine, Institute of Respiratory Health, State Key Laboratory of Respiratory Health and Multimorbidity, Frontiers Science Center for Disease-related Molecular Network, Sichuan Provincial Engineering Laboratory of Precision Medicine, Precision Medicine Key Laboratory of Sichuan Province, West China Hospital, West China School of Medicine, Sichuan University, Chengdu 610041, Sichuan Province, China; 2 Department of Ophthalmology, Second Affiliated Hospital of Xi'an Jiaotong University, Xi'an 710004, China; 3 College of Biological Sciences and Engineering, Shaanxi University of Technology, Hanzhong 723005, China

## Abstract

Idiopathic pulmonary fibrosis (IPF), a progressive and fatal lung disease, significantly increases the risk of lung cancer, particularly lung adenocarcinoma (LUAD). However, the shared genetic mechanisms driving IPF and LUAD comorbidities remain poorly understood, necessitating integrated multi-omics investigations. Through bulk and single-cell transcriptomic analyses, we identified 308 shared differentially expressed genes enriched in lipid metabolism and immune-inflammatory processes. Additionally, single-cell profiling revealed significant alterations in epithelial cells and macrophage populations between LUAD and IPF tissues, underscoring their role in disease progression. Furthermore, the copy number variation profiling identified a premalignant epithelial subpopulation in IPF exhibiting transcriptional signatures resembling LUAD malignant epithelial cells, and trajectory analysis illustrated a potential temporal progression toward malignancy. To identify co-causal genes, we performed weighted gene coexpression network analysis, defining modules associated with key cell types involved in comorbidities. Moreover, leveraging 101 algorithm combinations across ten machine learning approaches, we constructed a robust prognostic model, pinpointing CHST6 as a top prognostic gene consistently upregulated in both LUAD and IPF. Functional validation confirmed that CHST6 promotes lung cancer cell proliferation, migration, and invasion. In conclusion, our findings elucidate the shared molecular landscape of LUAD and IPF and propose that CHST6 is a promising co-disease therapeutic target.

## INTRODUCTION

Idiopathic pulmonary fibrosis (IPF), a progressive and fatal interstitial lung disease of unknown etiology, is pathologically characterized by irreversible extracellular matrix (ECM) deposition and architectural distortion of lung parenchyma, ultimately leading to respiratory failure.^1,2^ The global burden of IPF continues to rise, with its clinical trajectory frequently complicated by significant comorbidities. Notably, IPF patients exhibit a three- to fivefold elevated risk of developing lung adenocarcinoma (LUAD) compared to the general population, with malignant lesions preferentially emerging in fibrotic peripheral lung zones.^3–5^ Epidemiological studies have revealed that 3%–22% of IPF patients develop LUAD, revealing a striking clinical association that strongly suggests fundamental mechanistic links between these conditions.^6^ Therefore, this compelling epidemiological synergy highlights the critical need to identify shared molecular pathways between IPF and LUAD pathogenesis.

Substantial evidence indicated that IPF and LUAD share common pathogenic mechanisms, including recurrent genetic alterations (e.g., TERT promoter mutations), epigenetic dysregulation, and aberrant activation of profibrotic and pro-oncogenic pathways.^7–9^ These pathologically distinct conditions converge on a similarly dysregulated microenvironment characterized by interconnected processes such as aberrant TGF-β signaling that drives stromal activation and ECM remodeling; PD-L1-mediated immune evasion that attenuates antitumor immunity and promotes disease progression;^10,11^ and progressive ECM stiffening that potentiates both fibrotic advancement and tumor invasion.^12,13^ Together, these pathological alterations establish a permissive niche for the coevolution of fibrosis and malignancy. However, the precise mechanistic transition from fibrotic stroma to malignant transformation remains incompletely understood, particularly regarding how the interplay between matrix stiffness, chronic inflammation, and epithelial–mesenchymal plasticity synergistically drives carcinogenesis within fibrotic niches.^3^ Moreover, the tumor microenvironmental components and their roles common to both IPF and LUAD have been insufficiently investigated. Systematic characterization of these intertwined molecular pathways is critical for elucidating the coevolutionary dynamics of IPF and LUAD and may yield transformative insights into disease mechanisms and pave the way for novel therapeutic strategies targeting both fibrotic and neoplastic processes.

Recent advances in single-cell technologies have revolutionized the understanding of the pathological microenvironments underlying both LUAD and IPF, revealing both convergent and disease-specific mechanisms driving pathogensis.^14–19^ In LUAD, the tumor microenvironment (TME) is orchestrated by immunosuppressive niches dominated by (a) dysfunctional T cell populations with exhausted phenotypes, (b) M2-polarized tumor-associated macrophages (TAMs) promoting immune evasion, (c) PD-1/PD-L1-driven myeloid suppression, and (d) cancer-associated fibroblasts (CAFs) that drive metastatic progression through TGF-β/IL-6-mediated ECM remodeling and angiogenic reprogramming.^14,20^ Similarly, IPF exhibits a “fibrotic niche” characterized by (a) profibrotic macrophage subsets sustaining chronic inflammation, (b) aberrant epithelial–mesenchymal crosstalk triggering aberrant repair, and (c) stromal hyperactivation through TGF-β and Wnt signaling.^15,21^ Both diseases exhibit metabolic symbiosis (e.g., hypoxia-induced lipid scavenging in LUAD) and pathogenic ligand–receptor interactions (EGFR/Notch in LUAD; dysregulated epithelial–stromal signaling in IPF), which collectively contribute to therapeutic resistance. While LUAD centers on immune evasion and tumor–stroma metabolic coupling, IPF progression is dominated by maladaptive wound healing and excessive ECM deposition. Emerging spatially resolved multi-omics approaches are bridging these microenvironmental paradigms, enabling precision therapeutic strategies—immune checkpoint blockade combined with stromal modulation for LUAD vs the targeted disruption of fibrogenic signaling loops in IPF. These single-cell insights are transforming microenvironment-focused therapeutics across both oncology and fibrotic diseases, highlighting the power of high-resolution molecular profiling in uncovering actionable biological nodes.^20,22^

In this study, we employed integrated multi-omics data to systematically investigate the molecular interplay between IPF and LUAD, with particular emphasis on characterizing their shared microenvironment alterations. Through comprehensive bioinformatics analyses of transcriptomic data from diseased and normal lung tissues, combined with advanced machine learning algorithms, we further identified the underlying pathogenic pathways and potential co-disease therapeutic targets. Our findings elucidate shared microenvironment remodeling features and core molecular networks of LUAD and IPF, as well as the promising co-disease therapeutic candidate CHST6 with dual efficacy.

## RESULTS

### Co-disease transcriptomic integration reveals shared DEGs and dual metabolic-immune dysregulation in LUAD and IPF

**FIG. 1.**
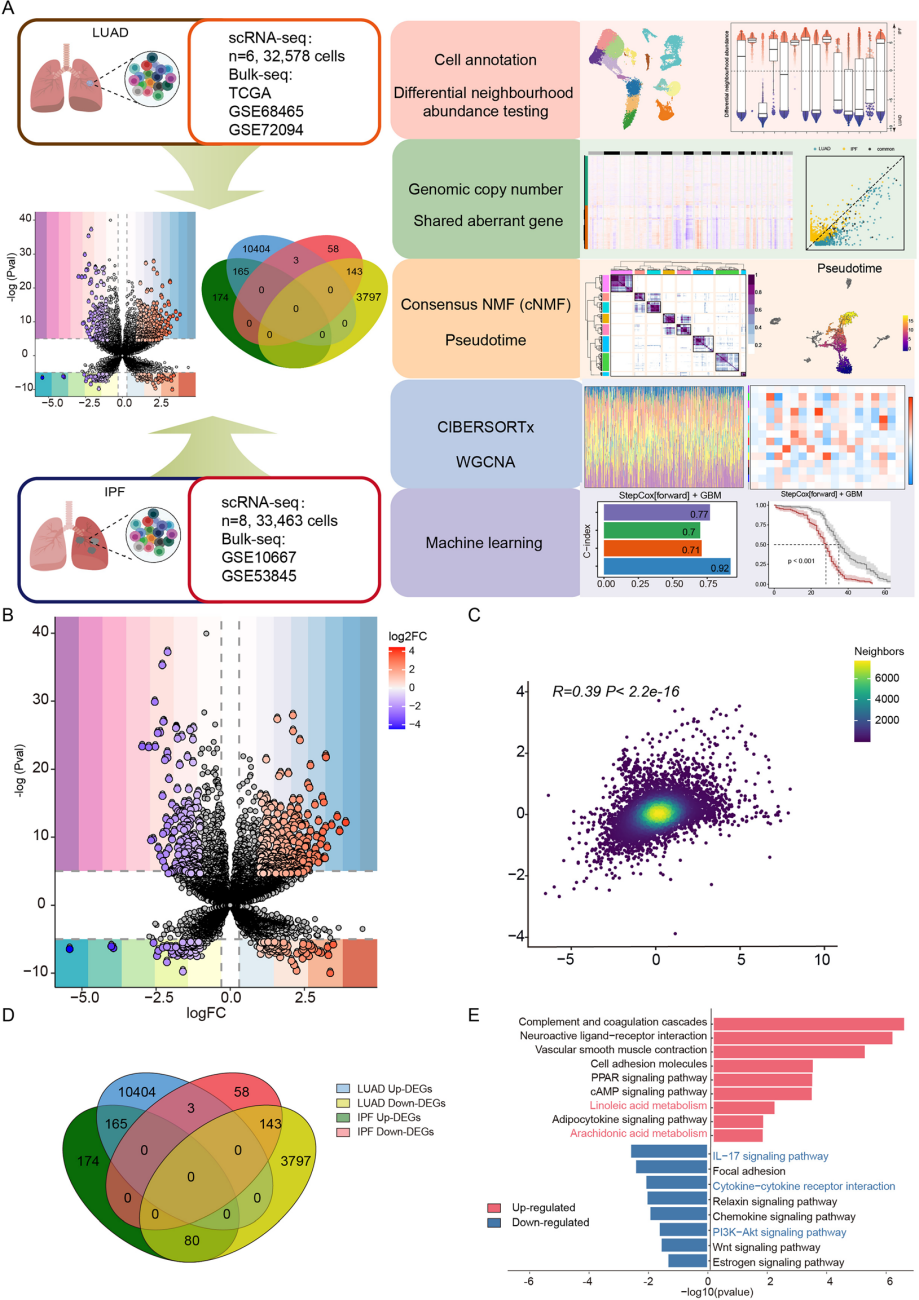
Co-disease transcriptomic integration reveals shared DEGs and dual metabolic-immune dysregulation in LUAD and IPF. (a) The workflow diagram illustrates the data analysis process. (b) Identification of common differentially expressed genes. Volcano map of DEGs in LUAD (top half). Volcano map of DEGs in IPF (bottom half). (c) Correlation analysis of differentially expressed genes in LUAD and IPF. (d) Venn diagram identifying co-upregulated and co-downregulated DEGs. (e) GSEA results showing the commonly upregulated DEGs whose expression was downregulated.

The analytical workflow is illustrated in Fig. 1(a). To explore potential pathogenic links between LUAD and IPF, we integrated transcriptomic data from the TCGA LUAD cohorts (all paired samples included) and two independent IPF datasets (GSE10667 and GSE53845).^23–25^ Differentially expressed genes (DEGs) were filtered using stringent thresholds (|log2FC| ≥ 1, adjusted p < 0.05), revealing 10 572 upregulated and 4020 downregulated genes in LUAD, compared with 419 upregulated and 204 downregulated genes in IPF [Fig. 1(b)]. An intersection analysis revealed 308 shared DEGs between the two diseases [Fig. 1(c)], suggesting convergent molecular mechanisms. The significant positive correlation (R = 0.39, p < 0.01) among these shared DEGs further supported their functional interplay in LUAD progression and IPF pathogenesis [Fig. 1(d)]. Pathway enrichment analysis demonstrated consistent upregulation of lipid metabolism pathways (linoleic and arachidonic acid metabolism) in both diseases, whereas downregulated pathways predominantly involved immune–inflammatory processes (e.g., cytokine–cytokine receptor interactions and chemokine signaling), highlighting the dual roles of metabolic dysregulation and immune suppression in disease development [Fig. 1(e)]. This multi-omics integration revealed 308 shared molecular signatures between LUAD and IPF, implicating synergistic roles of lipid metabolic activation and immunosuppression in their co-pathogenesis and identifying potential therapeutic targets for both diseases.

### Single-cell atlas reveals divergent microenvironment dynamics and conserved transcriptional hubs in LUAD and IPF tissues

**FIG. 2.**
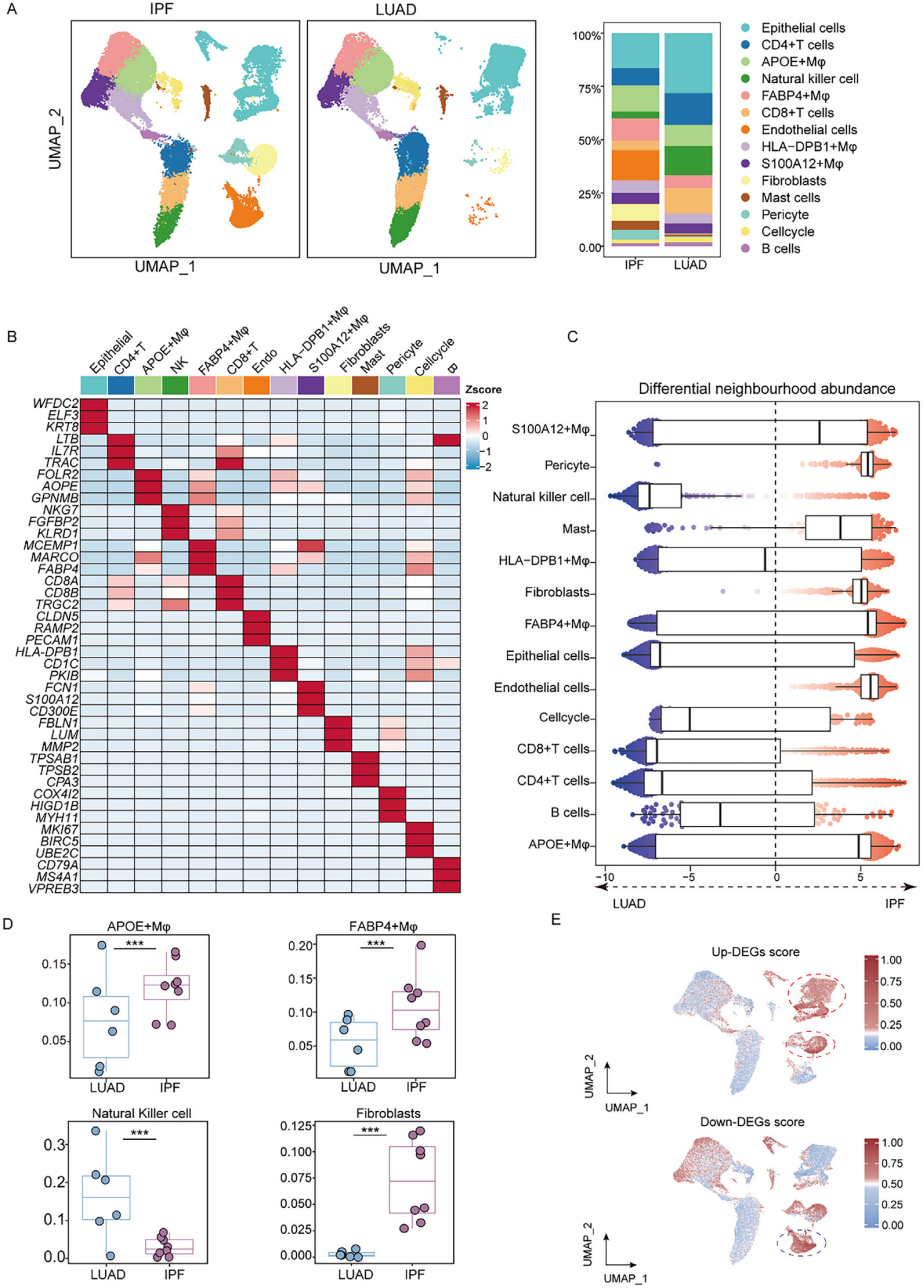
Single-cell profiling reveals distinct cell types and ploidy statuses within the analyzed population. (a) UMAP plot showing distinct cell types identified on the basis of canonical marker gene expression. (b) Heatmap displaying the top five highly expressed genes for each cell type. (c) Beeswarm plot illustrating differential cell abundance in LUAD, with annotated neighborhood clusters compared between LUAD and IPF. (d) Boxplots showing the cellular fractions of APOE+ macrophages, FABP4+ macrophages, natural killer (NK) cells, and fibroblasts in LUAD and IPF patients. The centerline indicates the median; boxes represent the interquartile range (IQR), and whiskers extend to 1.5× IQR. Each dot represents an individual sample. All comparisons showed adjusted p values >0.05. Statistical analysis was performed via one-sided unpaired Wilcoxon tests, with false discovery rate (FDR) correction. (e) Scores of commonly upregulated and downregulated DEGs at the single-cell level.

To further investigate the molecular mechanisms underlying the microenvironment at single-cell resolution in LUAD and IPF, we analyzed single-cell sequencing data from six LUAD tissues and integrated them with eight publicly available IPF datasets.^26,27^ To minimize batch effects, all datasets were harmonized via the Harmony algorithm, resulting in a total of 66 041 single cells, including 32 578 cells from LUAD and 33 463 from IPF. Cell clusters were identified via Seurat's graph-based clustering algorithm, followed by dimensionality reduction and visualization via uniform manifold approximation and projection (UMAP) [Fig. 2(a)]. On the basis of lineage-specific marker expression, we identified 14 principal cell populations: epithelial cells (*ELF3*, *KRT8*), CD4+ T cells (*LTB*, *IL7R*), APOE+ macrophages (*FOLR2*, *APOE*), natural killer (NK) cells (*FGFBP2*, *NKG7*), FABP4+ macrophages (*MARCO*, *FABP4*), CD8+ T cells (*CD8A*, *CD8B*), endothelial cells (*CLDN5*, *PECAM1*), HLA-DPB1+ macrophages (*HLA-DPB1*, *CD1C*), S100A12+ macrophages (*S100A12*, *FCN1*), fibroblasts (*LUM*, *MMP2*), mast cells (*TPSAB1*, *CPA3*), pericytes (*COX4I2*, *HIGD1B*), cycling cells (*MKI67*, *BIRC5*), and B cells (*CD79A*, *MS4A1*) [Fig. 2(b)]. We further analyzed the proportions of cell types and found significant differences between LUAD and IPF. We performed fine-resolution clustering and annotation and applied the “miloR” tool to quantify shifts in the abundance of all cell types between LUAD and IPF.^28^ The results revealed significant differences in APOE+ Mφs, FABP4+ Mφs, NKs, and fibroblasts between LUAD and IPF patients [Fig. 2(c)]. To characterize the heterogeneity of the microenvironment in these two diseases, we calculated the fractions of different cell types in LUAD and IPF patients. We observed that the fractions of APOE+ Mφ, FABP4+ Mφ, and fibroblasts were increased in IPF patients, but that of NK was decreased in IPF patients [Fig. 2(d)]. The common DEG score was enriched mainly in epithelial cells and fibroblasts [Fig. 2(e)]. Single-cell deconvolution revealed divergent immune–stromal rewiring in LUAD and IPF, with epithelial cells, fibroblasts, and macrophage subsets emerging as central hubs for shared transcriptional programs, providing a cellular framework for targeting common pathogenic nodes.

### Reprogramming of lipid metabolism in epithelial cells drives the common pathogenesis of IPF and LUAD

**FIG. 3.**
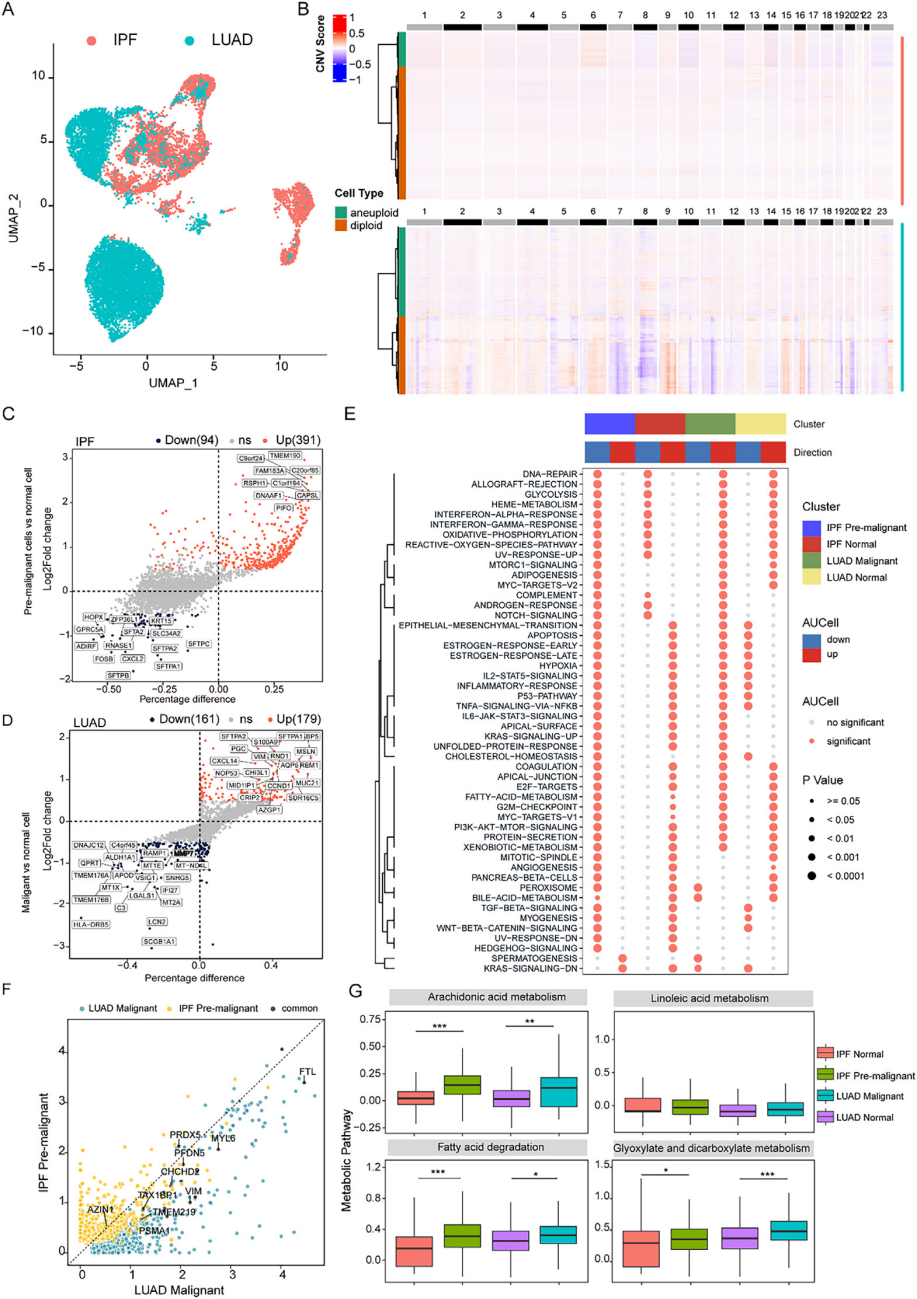
Reprogramming of lipid metabolism in epithelial cells drives the common pathogenesis of IPF and LUAD. (a) UMAP plot showing the distribution of epithelial cells from IPF and LUAD samples. (b) Inference of aneuploid tumor cells in LUAD and IPF samples via the CopyKAT algorithm. (c) Differentially expressed genes (DEGs) between premalignant and normal epithelial cells in IPF. (d) Differentially expressed genes (DEGs) between pmalignant and normal epithelial cells in LUAD. (e) Pathway enrichment analysis of upregulated and downregulated DEGs in IPF and LUAD. AUCell scores were used to evaluate pathway activity at the single-cell level. (f) Correlation analysis of DEGs between malignant and nonmalignant epithelial cells. (g) The scMetabolism analysis revealing heterogeneity in metabolic pathway activity between malignant and nonmalignant epithelial cells.

Both LUAD and IPF share persistent epithelial cell injury and dysfunction as central driving forces of disease progression. However, their downstream pathological phenotypes differ, leading to malignant proliferation in LUAD and fibrotic scar formation in IPF. To further validate the pathogenesis of LUAD and IPF with abnormal gene expression in epithelial cells, epithelial cell analysis involved identifying copy number variations (CNVs) via the “CopyKAT” package, which enables the classification of cells on the basis of ploidy status [Fig. 3(a)]. This analysis revealed 3331 aneuploid and 2137 diploid cells in IPF, as well as 4985 aneuploid and 4143 diploid cells in LUAD [Fig. 3(b)]. Notably, the aneuploid cell population was designated as tumor cells. Notably, we identified a minor population of pulmonary epithelial cells in IPF patients exhibiting histopathological features suggestive of early malignant transformation, characterized by distinct cytomorphological alterations despite a lack of significant CNV aberrations. These preneoplastic cells may constitute transitional precursors in the pathogenetic continuum linking pulmonary fibrosis to lung carcinogenesis. To delineate transcriptional dysregulation in pulmonary epithelial pathogenesis, we performed comparative transcriptomic profiling across three cellular states: normal epithelium, IPF-associated premalignant lesions, and LUAD tumors. The analysis revealed stage-specific signatures: (a) IPF premalignancy presented 391 upregulated genes (including *TMEM190*, *C9orf24*, and *FAM183A*) alongside 91 downregulated markers (e.g., *SFTPB*, *SFTPA*, and *CXCL2*); (b) LUAD progression presented 179 upregulated genes (notably *SFTPA1*, *SFTPA2*, and *S100A9*) associated with IPF premalignancy, with 161 unique suppressed genes (*SCGB1A1*, *LCN2*, and *HLA-DRB5*) [Figs. 3(c) and 3(d)]. Pathway enrichment analysis revealed significant activation of core oncogenic signaling axes, including epithelial–mesenchymal transition (EMT), apoptosis regulation, and hypoxia response pathways [false discovery rate (FDR) <0.05], in IPF-associated premalignant lesions. Notably, coordinated upregulation of fatty acid metabolic reprogramming was concurrently observed. This pathogenic signature demonstrated substantial overlap with the molecular mechanisms driving LUAD progression, particularly in the metabolic adaptation and survival signaling modules [Fig. 3(e)]. Intersection analysis of transcriptomic profiles between LUAD and IPF epithelial lineages revealed four conserved dysregulated genes (FDR <0.01, |logFC| > 1), *FTL*, *MYL6*, *PRDX6*, and *PFDN5*, with concordant expression patterns across both pathologies. Cross-disease correlation networks revealed that these genes constitute core regulatory nodes bridging fibrotic remodeling and neoplastic transformation [Fig. 3(f)]. The “scMetabolism” category revealed significant activation of four lipid-related pathways—arachidonic acid metabolism, linoleic acid metabolism, fatty acid degradation, and glyoxylate dicarboxylate metabolism—in malignant epithelial cells compared with their normal counterparts across both IPF and LUAD [Fig. 3(g)]. These findings demonstrate that conserved epithelial metabolic dysregulation is a shared pathogenic mechanism in fibrotic and neoplastic lung diseases, corroborating the bulk RNA-seq results.

### Transcriptional transition between the malignant meta-program shared by premalignant cells in IPF and malignant cells in LUAD

**FIG. 4.**
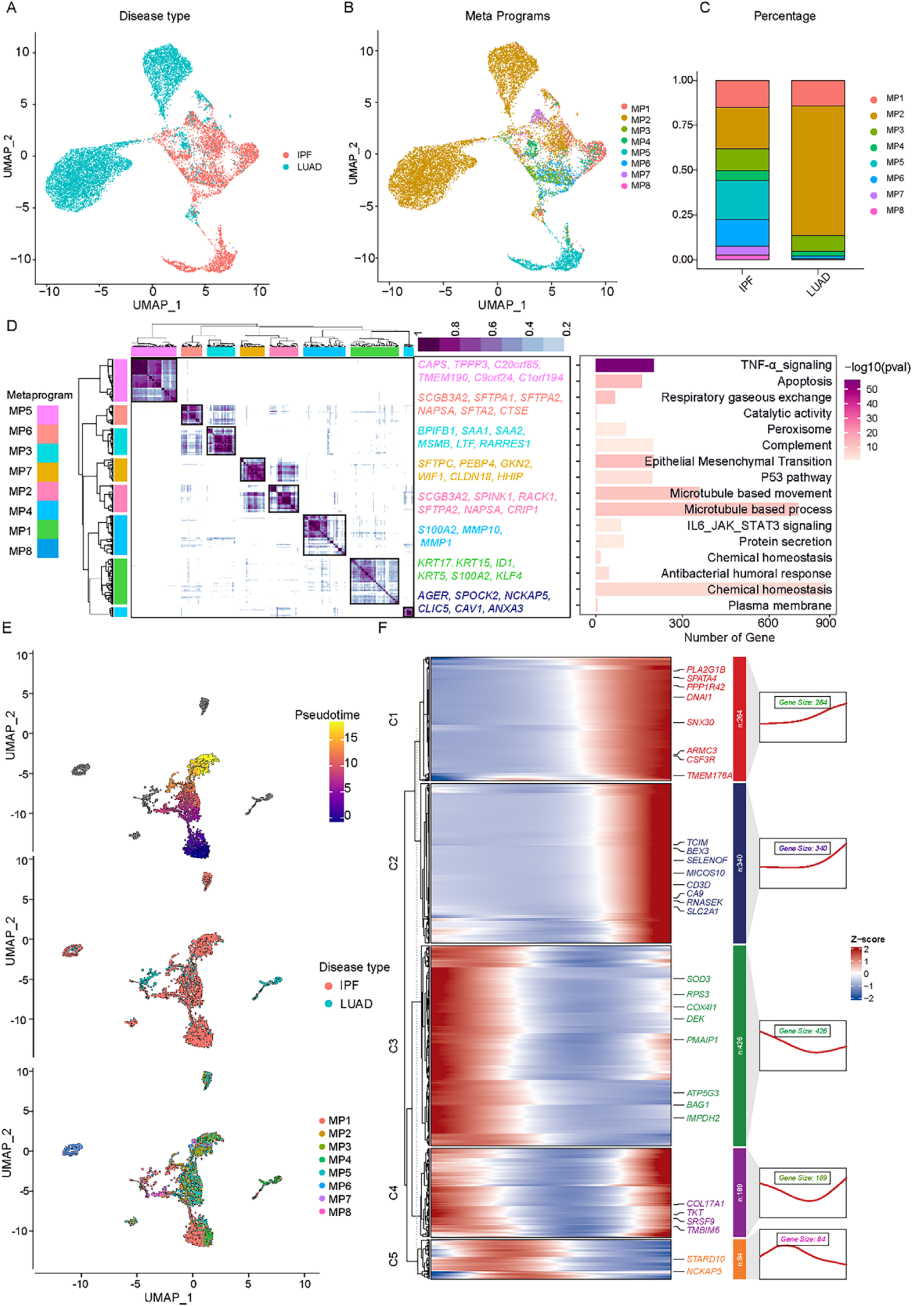
Identification of malignant cell-associated meta-programs between IPF and LUAD. (a) UMAP plot comparing the distribution of malignant cells between LUAD and IPF. (b) UMAP plot showing the distribution of eight non-negative matrix factorization (NMF) gene modules. (c) Distribution of gene modules in LUAD vs IPF, highlighting differences in module expression. (d) Heatmap depicting the correlation of all 88 signatures derived from the cNMF algorithm. Eight highly correlated expression programs are highlighted, along with the top enriched pathways associated with tumor expression programs. (e) Proportion of malignant cells in various states and pseudotime trajectories predicted by Monocle3. (f) Heatmaps showing gene expression and cellular components of the malignant cell module associated with pseudotime progression.

Owing to the high transcriptional specificity and heterogeneity of malignant cells within tumors, conventional clustering methods often fail to robustly categorize them into biologically meaningful subgroups. To gain deeper insights into the transcriptomic programs associated with intratumoral malignant cell heterogeneity, we applied consensus non-negative matrix factorization (NMF) to identify coherent gene sets preferentially coexpressed by individual cells across 17 tumors in the single-cell RNA sequencing (scRNA-seq) cohort [Fig. 4(a)]. This analysis yielded 88 gene signatures, which were subjected to hierarchical clustering to reveal 8 distinct transcriptomic meta-programs (MPs) with discrete biological functions [Fig. 4(b)]. We found that the meta-programs MP1, MP2, MP3, and MP4 are common to both LUAD and IPF [Fig. 4(c)]. MP1 is involved in the expression of TNF-α signaling genes (*KRT17*, *KRT15*, *ID1*, *KRT5*, *S100A2*, *KLF4*), and MP2 is characterized by the expression of respiratory gaseous exchange genes (*SCGB3A2*, *SPINK1*, *RACK1*, *SFTPA2*, *NAPSA*, *CRIP1*), which are activated by different stimuli and stress responses. MP3 was enriched in peroxisome genes (*BPIFB1*, *SAA1*, *SAA2*, *MSMB*, *LTF*, *RARRES1*), whereas MP4 was associated with epithelial–mesenchymal transition, with the upregulation of the *S100A2*, *MMP10*, *KRT17*, *KRT15*, *KRT5*, and *MMP1* genes, and the MP5 gene included microtubule-based movement-related genes (*CAPS*, *TPPP3*, *C20orf85*, *TMEM190*, *C9orf24*, *C1orf194*). MP6 includes IL6–JAK–STAT3 signaling-related genes (*SCGB3A2*, *SFTPA1*, *SFTPA2*, *NAPSA*, *SFTA2*, *CTSE*) and may be hypoxia dependent. MP7 includes chemical homeostasis-related genes (*SFTPC*, *PEBP4*, *GKN2*, *WIF1*, *CLDN18*, *HHIP*) and may be hypoxia dependent. MP8 included hypoxia-related genes (*S100A2*, *MMP10*, *MMP1*) and may be hypoxia dependent [Fig. 4(d)]. These results suggest that the shared presence of these transcriptional modules in both LUAD and IPF may play a role in disease progression. Next, we used Monocle3 simulations to establish the differentiation trajectories of cells between the common meta-programs, and trajectory analysis performed for malignant cells revealed the states and branches in the cell trajectory [Fig. 4(e)]. By identifying genes correlated with pseudotime and sorting them into three distinct categories—C1, C2, C3, C4, and C5—functional assessments of these genes were conducted via GO enrichment analysis, as depicted in Fig. 4(f). This result reveals a potential temporal trajectory for the transformation of the shared malignant program.

### Screening for key modules with the highest positive correlation to disease states via WGCNA

**FIG. 5.**
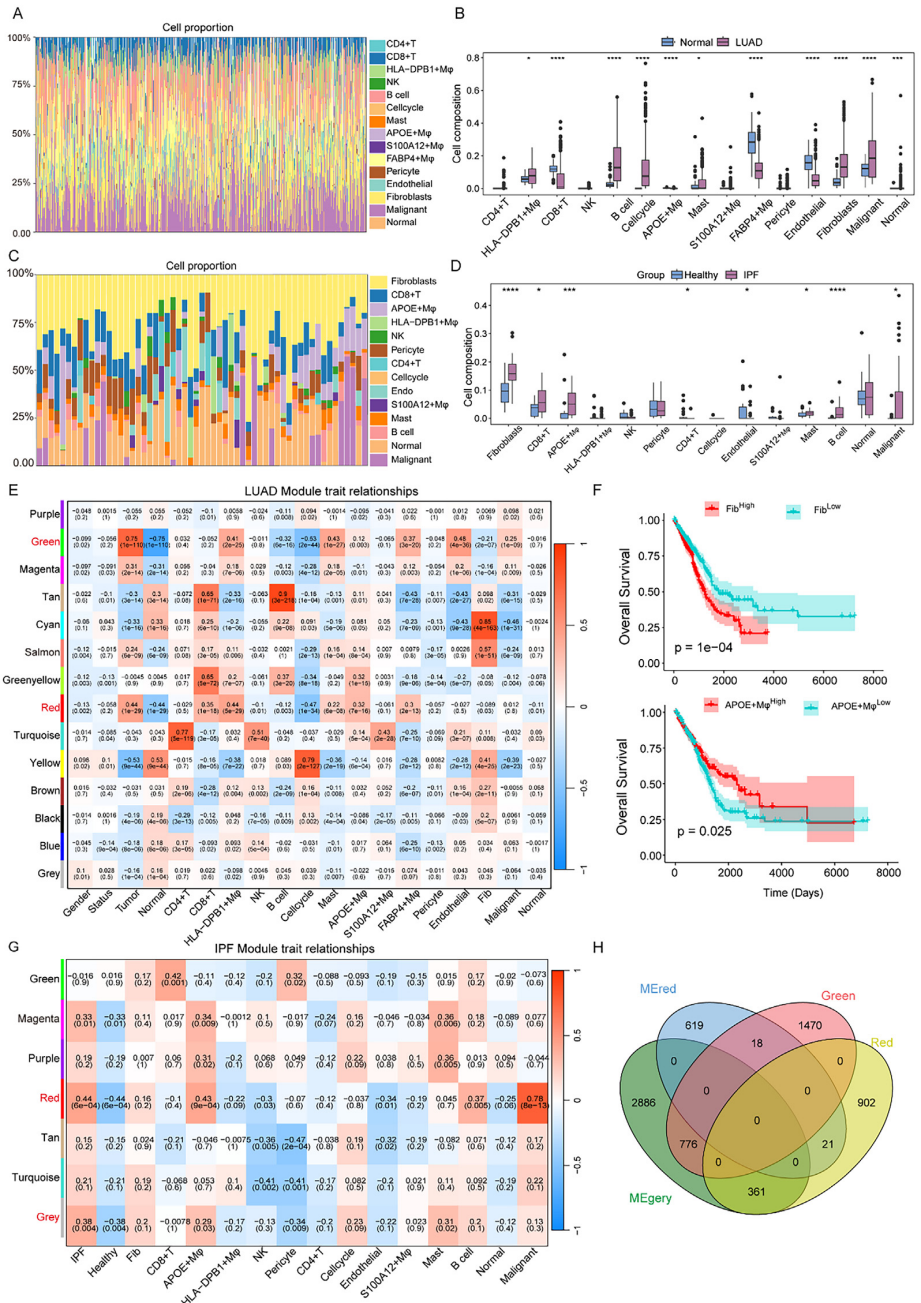
Screening for key modules with the highest positive correlation to disease states via WGCNA. (a) Reverse convolution analysis of LUAD bulk mRNA-seq data via the “CIBERSORTx” algorithm to construct the feature matrix. (b) Relationships between gene modules and traits in LUAD, with correlation coefficients and p values provided for each cell. (c) Differences in the distribution of cell types between LUAD and normal tissues. (d) Reverse convolution analysis of IPF bulk mRNA-seq data via the “CIBERSORTx” algorithm to construct the feature matrix. (e) Relationships between gene modules and traits in IPF, with correlation coefficients and p values provided for each cell. (f) Differences in the distribution of cell types between IPF and normal tissues.

Next, we employed CIBERSORTx, a deconvolution algorithm, to analyze the cellular changes associated with LUAD and IPF on the basis of single-cell transcriptomic data. Using the LUAD single-cell dataset as a reference, we applied the CIBERSORTx cell-type deconvolution method and the Wilcoxon test to compare the distributions of 15 cell types across different LUAD molecular subpopulations [Fig. 5(a)]. Notably, we observed a significant increase in HLA-DPB1+ Mφ, B cells, cycling cells, fibroblasts, and malignant cells in LUAD. Conversely, the numbers of CD8+ T cells, FABP4+ macrophages, and endothelial cells were reduced in LUAD and IPF tissue [Fig. 5(b)]. Using the same approach, we employed the IPF single-cell dataset as a reference to deconvolute the proportions of 11 cell types in IPF. The analysis revealed a significant increase in fibroblasts, APOE+ Mφ, B cells, and malignant cells in fibrotic tissue [Figs. 5(c) and 5(d)]. To further explore key gene modules associated with LUAD, we performed weighted gene coexpression network analysis (WGCNA). The optimal soft-thresholding powers for constructing the scale-free network were 11, 10, and 6. As shown in Fig. 3(b), hierarchical clustering was performed via the TOM dissimilarity measure. We identified 14 coexpression modules in total. The modules for which p < 0.05 were regarded as key modules. As shown in Fig. 3(c), the Green Salmon Red module had the strongest positive correlation, which included 2264 genes [Fig. 5(e)]. To further validate the impact of the ratio of fibroblasts to APOE+ macrophages on LUAD survival, we performed survival analyses, which revealed that patients in the high-infiltration group had significantly shorter survival times than those in the low-infiltration group did [Fig. 5(f)]. Additionally, WGCNA was applied to the IPF group, and b = 10 was the optimal value for soft power. We identified seven modules in total, in which red and gray were strongly positively correlated [Fig. 5(g)]. From the two key modules identified in the IPF group, we further selected 4023 genes. These genes, together with those from the corresponding key modules in both groups, may serve as potential candidate markers specific to certain cell types. In summary, we identified two gene modules in LUAD and IPF that are associated with disease onset, and the combined gene sets from these modules may represent key contributors to the shared pathogenic mechanisms underlying both cell types [Fig. 5(h)].

### Construction and validation of an integrin-related signature in osteosarcoma via machine learning algorithms

**FIG. 6.**
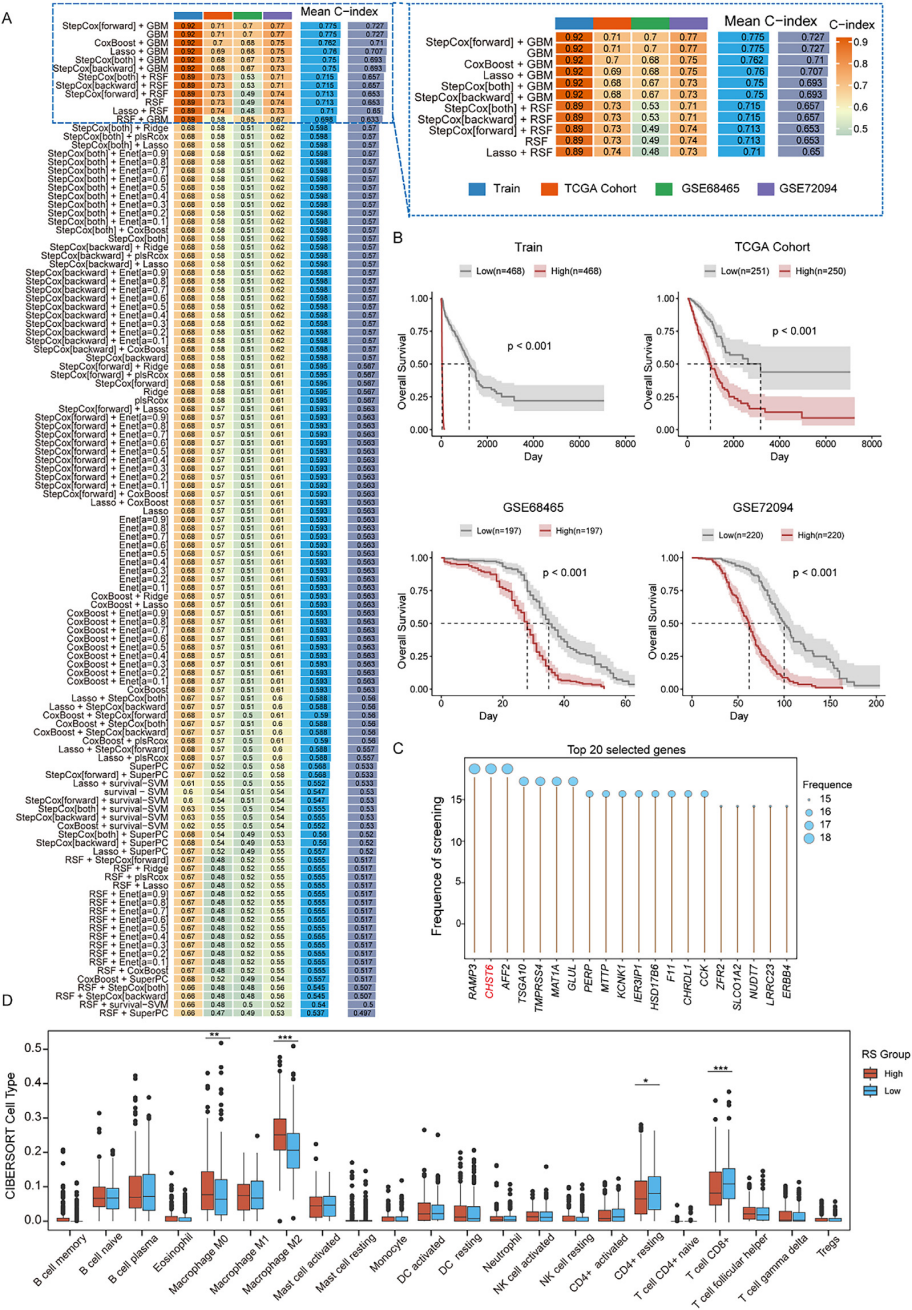
Assessment of prognostic gene-based models and survival risk factors. (a) A total of 101 prediction models were developed across a tenfold cross-validation framework, and the C-index for each model was calculated for all the validation datasets. (b) Survival curves based on the risk model constructed with the training dataset, TCGA cohort, GSE68465 cohort, and GSE72094 cohort. (c) Screening and ranking of the top 20 genes based on the risk model. (d) Immune cell infiltration analysis based on the risk model using the TCGA dataset and CIBERSORT algorithm, showing the proportions of immune cell types in LUAD samples.

We applied a suite of 101 machine learning algorithms to analyze the total set of candidate genes. The results from these models revealed that the top 12 ranked by the average C-index consistently employed gradient boosting machine (GBM), StepCox, random survival forest (RSF), or various parameterized combinations of these algorithms. Notably, the area under the curve (AUC) for most models exceeded 0.9, with the top three models achieving an average C-index above 0.76 [Fig. 6(a)]. Owing to its simplicity and robustness, we selected the backward selection method for subsequent validation. Patient survival was further evaluated across multiple datasets, which revealed that the high-risk group had a significantly worse prognosis than the low-risk group did (p < 0.01) [Fig. 6(b)]. The top 20 risk genes, ranked by importance, were *RAMP3*, *CHST6*, *AFF2*, *TSGA10*, *TMPRSS4*, *MAT1A*, *GLUL*, *PERP*, *MTTP*, *KCNK1*, *IER3IP1*, *HSD17B6*, *F11*, *CHRDL1*, *CCK*, *ZFR2*, *SLCO1A2*, *NUDT7*, *LRRC23*, and ERBB4 [Fig. 6(c)]. These findings indicate that elevated expression of these genes is associated with a greater risk of poor survival, underscoring a significant link between gene expression levels and survival outcomes and highlighting their potential as prognostic biomarkers. To further characterize the microenvironment in the high- and low-risk groups, we deconvoluted the bulk transcriptome with the help of the “CIBERSORT” algorithm. The results revealed that the proportions of M0 and M2 macrophages were significantly greater in the high-risk group than in the low-risk group, whereas the proportions of resting CD4^+^ T cells and CD8^+^ T cells were greater in the low-risk group [Fig. 6(d)]. These findings suggest that the high-risk group is associated with a more immunosuppressive tumor microenvironment, which is consistent with our previous analyses indicating that patients in the high-risk group have a poorer prognosis.

### The CHST6 gene may act as a common pathogenic factor in the LUAD

**FIG. 7.**
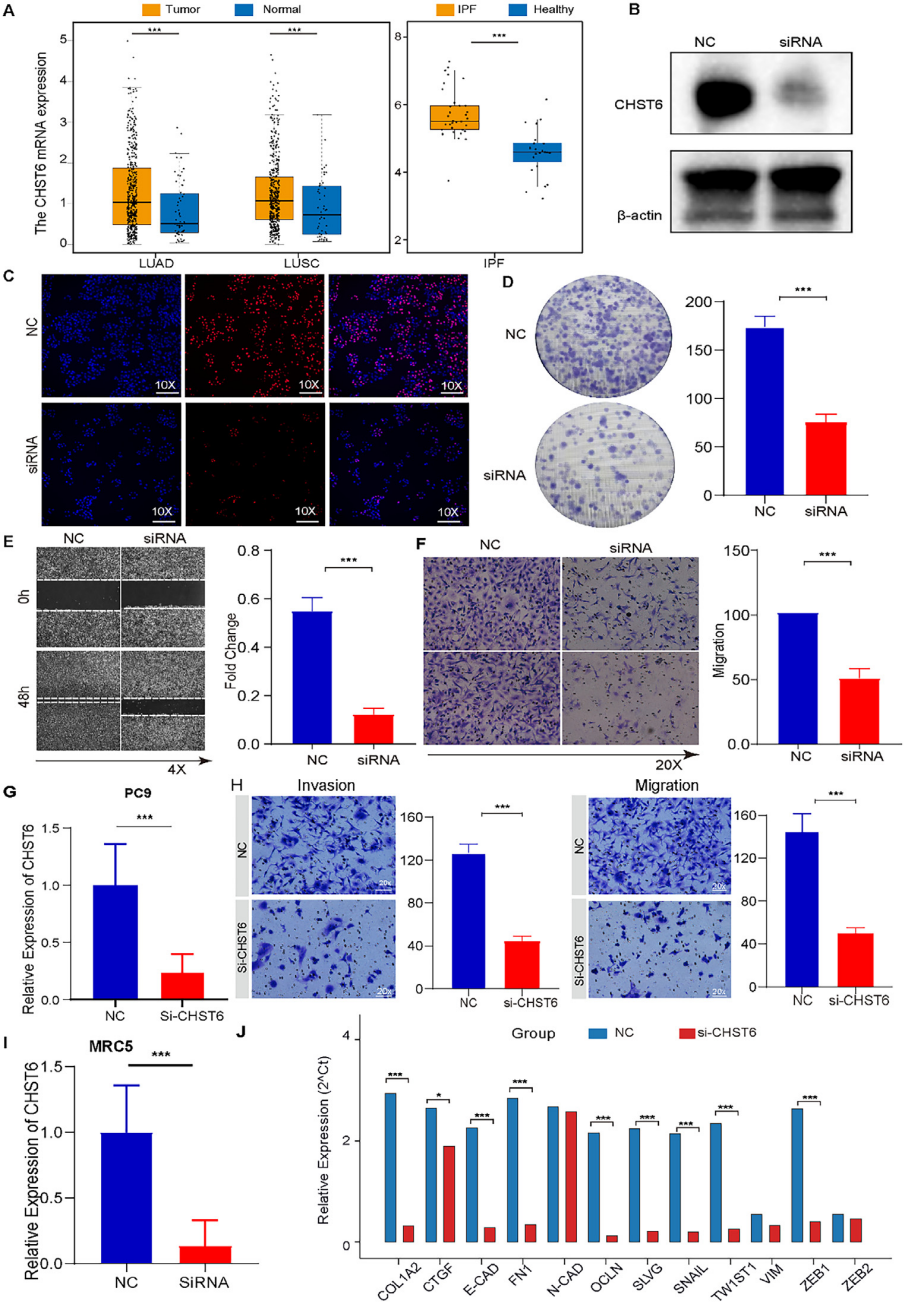
CHST6 drives enhanced proliferation and metastatic potential in IPF and LUAD. (a) The expression of the CHST6 gene in non-small cell lung cancer (NSCLC) patients was analyzed via the TCGA dataset, which was used to compare tumor tissues with normal tissues. Additionally, CHST6 expression was examined in idiopathic pulmonary fibrosis (IPF) patients by comparing healthy and diseased samples. (b) Western blot analysis confirmed the effect of CHST6 knockdown. (c) An EdU cell proliferation assay was used to evaluate cell proliferation after siRNA transfection. (d) Silencing CHST6 significantly suppressed the migratory and invasive capacities of lung cancer cells *in vitro*. (e) Cell migration was evaluated via a wound healing assay. The wound closure area was measured 48 h after scratch formation via ImageJ software. (f) Cell invasion was determined via the Transwell invasion assay, and the number of invasive tumor cells was quantified via ImageJ. (g) and (h) CHST6 knockdown in PC-9 cells significantly reduced migration and invasion compared with control cells. (i) and (j) CHST6 silencing in MRC-5 fibroblasts downregulated EMT/fibrosis genes (ZEB1, ZEB2, VIM, TWIST1, SNAIL, FN1, N-CAD, E-CAD, CTGF, and COL1A2).

CHST6, identified as a top-ranked candidate gene from integrated analyses, encodes a sulfotransferase involved in modifying heparan sulfate, which regulates cell adhesion, signaling, and immune response. Compared with that in healthy tissue, its expression was significantly elevated in both non-small cell lung cancer [NSCLC; LUSC (lung squamous cell carcinoma) and LUAD] and IPF tissues [Fig. 7(a)]. Next, total RNA was extracted from A549 cells, which were subsequently selected for further experiments [Figs. 7(b) and 7(c)]. The results revealed that CHST6 knockdown (si-CHST6 group) significantly reduced cell proliferation compared with the negative control starting at 48 h (p < 0.01), indicating that CHST6 promotes tumor cell growth [Fig. 7(d)]. Scratch wound healing assays demonstrated that CHST6 enhances the migratory capacity of lung cancer cells (p < 0.01 for A549 cells). In contrast, CHST6 inhibition significantly delayed wound closure in A549 cells (p < 0.01), suggesting impaired migration [Fig. 7(e)]. Furthermore, Transwell assays revealed that CHST6 knockdown markedly suppressed both the migration and invasion abilities of A549 cells [Fig. 7(f)]. To extend beyond A549 cells, we performed CHST6 knockdown experiments in the PC-9 lung adenocarcinoma cell line. siRNA-mediated CHST6 silencing markedly reduced CHST6 levels in PC-9 cells, and functional assays showed that CHST6 knockdown significantly impaired PC-9 cell migration and invasion compared with control cells [Figs. 7(g) and 7(h)], supporting a role for CHST6 in promoting LUAD cell aggressiveness. In addition, we used the MRC-5 lung fibroblast cell line to assess the effect of CHST6 knockdown on fibrosis-associated EMT markers. Silencing CHST6 led to a significant downregulation of EMT-related genes, including ZEB1, ZEB2, VIM, TWIST1, SNAIL, FN1, N-CAD, E-CAD, CTGF, and COL1A2, indicating that CHST6 may contribute to fibroblast activation and EMT processes in IPF [Figs. 7(i) and 7(j)].

## DISCUSSION

This study presents a systematic multi-omics investigation of the pathogenic interplay between LUAD and IPF, revealing their shared molecular landscape. By integrating multi-omics data, we identified several shared differentially expressed genes (DEGs), highlighting metabolic reprogramming and immune dysfunction as core pathogenic features under both conditions. Moreover, the scRNA-seq analysis further revealed the unique and overlapping characteristics of the microenvironment and fibrotic niches and demonstrated key signaling nodes driving disease progression in both conditions. These findings not only elucidate the mechanistic links between IPF and LUAD pathogenesis but also reveal novel therapeutic strategies for targeting their shared disease pathways.

Our study highlights critical molecular and cellular mechanisms underlying the pathogenic convergence of IPF and LUAD. Through integrated transcriptomic analysis, we identified a group of DEGs predominantly involved in dysregulated lipid metabolism and immunosuppressive pathways, emphasizing the synergistic role of metabolic reprogramming and immune evasion in disease progression. The lipid metabolism alterations demonstrate particular significance in both diseases. In IPF, metabolic dysregulation affects key metabolic pathways, including fatty acid oxidation and synthesis, cholesterol homeostasis, arachidonic acid derivative metabolism, and phospholipid remodeling during disease onset and development.^29,30^ Meanwhile, emerging evidence suggests that lipid metabolic abnormalities are equally critical in LUAD pathogenesis, particularly during early tumor initiation, premalignant lesion formation, and TME establishment.^31–35^

Additionally, single-cell analysis further revealed significant alterations in epithelial cells and macrophage populations in both LUAD and IPF tissues compared to normal tissues, underscoring their role in disease progression. These findings are consistent with previous studies of increased macrophage infiltration in both IPF and LUAD.^36,37^ Moreover, CNV analysis of IPF-derived epithelial cells identified a subpopulation exhibiting premalignant features resembling LUAD malignant cells, suggesting that chronic fibrotic remodeling may foster a microenvironment conducive to oncogenic transformation. Meanwhile, malignant epithelial cells in both diseases undergo metabolic reprogramming, particularly in fatty acid metabolism, corroborating the findings from bulk RNA-seq data. Our study also underscores the intratumoral heterogeneity of malignant cells, with NMF revealing distinct gene expression programs within LUAD tumors. Notably, several of these programs are associated with hypoxia and EMT, emphasizing the critical role of the microenvironment in disease progression.

WCGNA analysis identified critical gene modules significantly correlated with disease states, underscoring the pivotal role of epithelial cell, fibroblast, and macrophage interactions in shaping the microenvironment of LUAD and IPF. These findings deepen the understanding of the tumor microenvironment and its potential contribution to disease progression. To systematically explore shared molecular drivers, we developed a prognostic model based on an advanced machine learning framework incorporating 101 algorithm combinations across ten distinct approaches. Among the identified candidates, CHST6 emerged as a top prognostic gene, showing consistent upregulation in both LUAD and IPF. Recent studies suggest that CHST6 contributes to chronic lung disease pathogenesis by regulating mucin glycosylation and exhibiting sex-specific genetic associations in cystic fibrosis (CF) and chronic obstructive pulmonary disease (COPD).^38,39^ Further functional validation confirmed that CHST6 promotes lung cancer cell proliferation, migration, and invasion, suggesting its functional relevance in tumor biology. Collectively, these results highlight CHST6 as a potential cross-disease therapeutic target and provide novel insights into the molecular landscape of IPF and LUAD.

This study has several limitations. First, the current conclusions are mainly supported by *in vitro* assays and a limited number of *in vivo* experiments, which may not fully recapitulate the complexity of disease progression in human tissues. Second, the sample size and disease spectrum analyzed remain relatively small, which may restrict the generalizability of our findings. Third, although CHST6 was identified as a molecule of interest across diseases, its mechanistic contribution to disease pathology and therapeutic responsiveness has not been fully elucidated. Therefore, while CHST6 may represent a potential therapeutic target, further validation in diverse preclinical models and independent clinical cohorts is required to substantiate its cross-disease therapeutic relevance.

In conclusion, this study systematically demonstrates the pathological convergence between LUAD and IPF, underscoring the necessity of an integrated approach to unravel their complex interplay. Our findings reveal that shared molecular pathways such as lipid metabolic reprogramming are central drivers of disease progression in both IPF and LUAD and resemble cellular microenvironments and signaling networks in fibrotic and neoplastic niches. In addition, CHST6 identified in this study and other core mediators may present new opportunities for therapeutic intervention for dual efficacy. Although our single-cell transcriptomic analysis is comprehensive, it is constrained by dataset availability, which may limit its ability to fully capture patient heterogeneity across different disease stages. By elucidating the molecular crosstalk between these conditions, our study offers novel insights into their interconnected mechanisms and enables precision medicine strategies for these comorbid conditions.

## METHODS

### Data collection and processing

In our study, we conducted single-cell RNA sequencing (scRNA-seq) analysis on six freshly resected LUAD tissue samples via the 10× Chromium Genomics protocol, alongside an additional eight patient samples obtained from GSE128033, all of which were processed via the 10X Genomics platform (available at http://www.ncbi.nlm.nih.gov/geo/). Tissue samples were collected from patients at West China Hospital (WCH) who had been pathologically diagnosed with lung cancer and met the study's inclusion criteria. Patients were classified as having either *in situ* or invasive tumors on the basis of their pathological subtype. The study was conducted in compliance with the ethical guidelines of the West China Hospital Ethics Committee.

To investigate transcriptional differences, we integrated three independent bulk RNA-seq datasets of idiopathic pulmonary fibrosis (IPF) from the GSE10667 and GSE53845 datasets. Additionally, we obtained bulk RNA sequencing data and corresponding clinical information for LUAD from the TCGA database, encompassing 501 samples with survival data, which were used for training our machine learning models. To validate our machine learning findings, we further analyzed 440 bulk RNA-seq datasets from GSE68465 and an additional 394 bulk RNA-seq datasets with survival information from GSE72094.

### Library preparation and sequencing

The tissue samples were rinsed with HBSS, minced on ice in HBSS with collagenase I/IV, and digested at 37 °C for 30 min with intermittent manual shaking. The suspension was centrifuged (500 × g, 5 min), and the pellet was resuspended in red blood cell lysis solution, followed by washing with HBSS. Single-cell preparation was performed via the Chromium Single-Cell 3′Gel Bead, Chip, and Library Kit v3 (10X Genomics) and the Chromium Gene Expression Solution. After cell lysis and RNA barcoding, the cells were encapsulated in emulsion gel beads, followed by cDNA amplification, fragmentation, and adapter/index attachment. Libraries were sequenced on the Illumina NovaSeq-6000 platform at West China Hospital.

### Analysis of single‐cell RNA sequence data

During the reprocessing of our scRNA-seq data, sequencing reads were first mapped to the GRCh38.p13 reference genome via CellRanger (v5.1.0), following standard procedures for sequence alignment, barcode assignment, and gene expression quantification. To ensure data integrity, we employed Seurat (4.4.0) for quality control and Harmony to correct for batch effects across samples.^26,27,40^ Cells were excluded if they met any of the following criteria: detection of fewer than 300 genes, mitochondrial gene content exceeding 20%, hemoglobin gene content over 5%, or genes expressed in fewer than three cells.

Beginning with normalization and scaling of expression matrices via the NormalizeData and ScaleData functions. To identify the most informative genes, we applied the FindVariableFeatures function, selecting the top 2000 highly variable genes for principal component analysis (PCA). Clustering was performed via the FindClusters function with the first 20 principal components and a resolution of 0.8, followed by manual verification to ensure cluster accuracy. Visualization was conducted via uniform manifold approximation and projection (UMAP). To define the 11 major cell types, we performed an initial exploratory analysis of the DEGs in each cluster, and the DEGs were identified via the “FindAllMarkers” function.

### Identification of malignant epithelial cells

The CopyKAT tool in R was utilized to assess cell-level copy number variations (CNVs), categorizing cells as either diploid or aneuploid.^41^ Cell clusters were subsequently reanalyzed to distinguish malignant cells from other cell types on the basis of their CNV profiles.

### Identification of differential gene expression (DEGs) via mRNA-seq

Before performing the biological analyses, we evaluated batch effects in the collected datasets and detected significant differences between the two diseases [Figs. 2(a) and 2(e)]. To eliminate these discrepancies and ensure robust analytical results, we applied the Combat package for batch effect correction. Differentially expressed genes (DEGs) were then identified via the LIMMA package, with significance thresholds set at p < 0.05 and |log_2_FC| > 1.

### Pseudotime and trajectory analysis

The malignant epithelial cell module uses Monocle3 (v2.14.0) for single-cell pseudo-temporal and trajectory analysis.^42^ Evolutionary processes were modeled as potentially discontinuous trajectories via the “learn_graph” function. Pseudotime ordering was performed with the order_cells function, where selected nodes were designated developmental starting points. Finally, genes exhibiting differential expressions along these trajectories were identified.

### WGCNA for coexpression network construction

Weighted gene coexpression network analysis (WGCNA) was employed to identify coexpressed gene modules, explore the relationships between gene networks and phenotypes, and determine core genes within the network. The WGCNA-R package was used to construct coexpression networks for all genes in the dataset, and the top 10 000 genes with the highest variance were selected for further analysis. To assess network connectivity, the weighted adjacency matrix was transformed into a topological overlap matrix (TOM), and a hierarchical clustering approach was applied to construct a clustering dendrogram. The branches of the dendrogram represent distinct gene modules, with different colors indicating different modules.^43^ Genes were categorized on the basis of their expression patterns, grouping functionally similar genes into the same module. Ultimately, tens of thousands of genes were classified into several modules according to their weighted correlation coefficients.

### Cell-type deconvolution via CIBERSORTx

To estimate the cellular composition of the bulk RNA-seq samples, we utilized CIBERSORTx, a high-resolution computational framework for cell-type deconvolution. A single-cell transcriptomic dataset was employed as a reference to define gene expression signatures for distinct cell populations. Initially, high-quality scRNA-seq data were preprocessed by filtering out low-quality cells and normalizing the expression levels. Cell type-specific expression profiles were then derived from the dataset to construct the reference matrix. CIBERSORTx (https://cibersortx.stanford.edu/) was run in “high-resolution” mode with 1000 permutations, allowing for batch effect correction and estimation of cell-type fractions in each bulk RNA-seq sample. The deconvolution results were further validated through statistical analyses, including the Wilcoxon rank-sum test, to assess differences in cell-type proportions between experimental conditions.

### Survival analysis and predictive Kaplan–Meier curves

The division of the Target dataset, including both the external and internal validation groups and the datasets GSE21257 and GSE39055, into high- and low-risk categories was based on the median IRS risk score. Analysis of Kaplan–Meier (KM) survival curves, conducted with the “survminer” R package, was aimed at discerning significant survival outcome disparities between these risk groups (log-rank test, p < 0.05). Furthermore, the “timeROC” package was utilized to perform ROC curve analysis to evaluate the prognostic sensitivity and specificity of the IRS in predicting survival outcomes. To confirm our model's predictive accuracy across various tumor entities, we examined data from the TCGA database and assessed significant survival outcome differences [*OS* (overall survival)] between the high- and low-risk groups (log-rank test, p < 0.05).

### RNA interference

PGF-targeting siRNAs were purchased from Tsingke (Beijing, China) and transfected into lung cancer cells using Lipofectamine 2000 (Invitrogen, Shanghai, China). Scrambled siRNA served as a control. Knockdown efficiency was confirmed by Western blotting 48 h post-transfection.

### Transwell invasion assay

A549 cells (3 × 10^4^) were seeded in the upper chamber of Matrigel-coated Transwell inserts (8 *μ*m pore size) with serum-free medium. The lower chamber contained 10% FBS as a chemoattractant. After 48 h, invaded cells were fixed, stained with gentian violet, and counted under a microscope.

### Wound healing assay

Cells at ∼85% confluence were scratched with a 200 *μ*l pipette tip and washed with PBS. They were then cultured in medium with 2% FBS. Images were taken at 0 and 24 h to assess wound closure.

### EDU assays

AGS cells were cultured with EdU (10 μM, 3 h), fixed (4% polyformaldehyde, 30 min), permeabilized (0.5% Triton X-100), and stained via click chemistry (Elabscience® Kit). Nuclei were counterstained with DAPI. Proliferation rates were quantified via fluorescence microscopy and analyzed via an Olympus microscope.

## Data Availability

The data that support the findings of this study are available from the corresponding author upon reasonable request.

## NOMENCLATURE

CHST6: Carbohydrate Sulfotransferase 6
CNV: Copy number variation
GSVA: Gene Set Variation Analysis
IPF: Idiopathic pulmonary fibrosis
KEGG: Kyoto Encyclopedia of Genes and Genomes
LUAD: Lung adenocarcinoma
MPs: Meta-programs
scRNA-seq: Single-cell RNA sequencing
TME: Tumor microenvironment
